# PACADI: translation and adaptation of a Swedish-language version of the pancreatic cancer disease impact score

**DOI:** 10.1186/s13104-022-06199-6

**Published:** 2022-10-12

**Authors:** Thomas Andersson, Monika Fagevik Olsén, Micheline Al Nouh, Svein Olav Bratlie

**Affiliations:** 1 Department of Surgery, Institute of Clinical Sciences, Sahlgrenska Academy, Sahlgrenska University Hospital, University of Gothenburg, 413 45 Gothenburg, Sweden; 2 Department of Health and Rehabilitation, Institute of Neuroscience and Physiology, Sahlgrenska Academy, University of Gothenburg, Gothenburg, Sweden; 3 Sahlgrenska Academy, University of Gothenburg, Gothenburg, Sweden

**Keywords:** Pancreatic surgery, Patient reported outcome, PACADI

## Abstract

**Objective:**

The Norwegian pancreatic cancer disease impact score (PACADI) is a digitalized analogue questionnaire that assesses different disease-specific symptoms. There is a need of translations of it into other languages. Therefore, the aim of this article is to describe the translation process of a Swedish version of PACADI and present its validity to EORCT QLQ PAN26. The self-administered questionnaire PACADI was translated according to guidelines and assessed by an expert panel of health care personnel. The test of its validity was performed with the disease-specific questionnaire for EORCT QLQ PAN26. Both questionnaires were completed by 66 subjects with pancreatic cancer, either before, at discharge or three months after surgery.

**Result:**

The results between the groups indicate that patients suffer from different symptoms at different times. The correlations between the different symptoms of the two questionnaires were fair to good. In conclusion, PACADI and QLQ PAN 26 have a good correlation and PACADI can be used in clinical practise.

## Introduction

Pancreatic cancer (PC) is an aggressive form of malignancy and one of the leading causes of cancer-related deaths [1]. Curative treatment, including surgical resection and adjuvant chemotherapy, is only feasible in about 20% of the patients due to severe co-morbidity, metastatic disease, or locally advanced tumor growth. Considering the severity of the disease, as well as treatment burden, patient reported outcome measures (PROM) is an important way to increase person-centeredness and better understanding of the effect of treatment [2, 3].

EORCT QLQ C-30 [5] and QLQ PAN26 [6] are often used to evaluate quality of life (QoL) in patients with PC. These are well known and used extensively in research. In a recently published review QLQ-PAN26 in conjunction with QLQ-C30, was recommended for exploring quality of life in patients with resectable pancreatic cancer [4]. However, considering barriers when using PROM, such as time required to complete the instrument [5], e.g. EORCT QLO-30 and QOL PAN26 comprises of 56 questions, it is necessary to find shorter alternatives.

A new questionnaire “The pancreatic cancer disease impact score” (PACADI) has been developed in Norway [6]. PACADI is a patient-derived, disease-specific digital instrument focusing on symptoms related to this specific kind of cancer. PACADI consists of eight symptom-variables which the patients assess on a visual analogue scale chart. To fully focus on symptoms of importance for the patients, groups of individuals with PC were involved in the development process. Preliminary psychometrical testing indicated a strong correlation with Euro Qol-5D (EQ5-D) index and Edmonton Symptom Assessment Scale (ESAS) “sense of wellbeing”. Also, PACADI demonstrated a high internal consistency as well as high test–retest reliability. [7]. As previous studies indicate PACADI to be a reliable and valid questionnaire, there is a rationale to translate it into other languages, such as Swedish, and psychometrically test it in clinical and research practice. Therefore, the aim of this article is to describe the translation process of a Swedish version of PACADI and present its validity to EORCT QLQ PAN26.

## Main text

### Materials and methods

The translational process of the Norwegian PACADI-version into Swedish was performed according to guidelines presented by Wild et al. [8]. Two independent health care professionals, both born in Sweden with experience of working in the surgical field in Norway made the translation into the Swedish language. A pooled version, reconciliation, was put together. Back translation into Norwegian was performed by two other health care professionals both born in Norway with several years’ experience of working in the surgical field in Sweden. Both versions were pooled, and a new version was put together. The Swedish version was compared with the original Norwegian version, and minor adjustments were made.

The second part of the study included 66 unique patients, 40 men and 26 women. These were recruited from surgical departments in three University hospitals in Sweden, Sahlgrenska University hospital in Gothenburg, Linköping University hospital and Uppsala University hospital. The Swedish version of PACADI and QLQ PAN26 was distributed to the patients before, at discharge or three months after surgery. Each patient responded once. The patients were asked to rate their experiences of the eight disease specific symptoms, called dimensions, during the past week.

The PACADI score (from 0 to 1) was calculated by summarizing each weighted value.

Face validity of the preliminary version was established through a review by 5 researchers, three nurses and two surgeons with clinical and scientific expertise in the field of pancreatic surgery. The patients were also asked to write comments on the relevance, description, and response options for the items in PACADI.

The EORTC QLQ-PAN26 is a supplementary questionnaire module to be employed in conjunction with the QLQ-C30, but as this trial´s aim was to compare disease specific questions only, the module for pancreatic cancer was used. Patients in the current study circled the number which indicated the extent to which they experienced each symptom or problem during the past week. Raw scores for each multi-item scale were calculated on the average of the corresponding items according to the PAN26 scoring guideline [9]. To be able to standardize the raw score to a 0–100 range a transformation was performed ((Raw score 1)/range*100).

In PACADI, one question characterizes each symptom, in contrast to QLQ PAN26, where each symptom is assessed by one or more questions. In this study, each question in PACADI, except for nausea, was analyzed to the relevant question(s) in QLQ PAN26 according to QLQ PAN26 scoring instructions (Table 1). The symptom nausea was removed as it does not have a corresponding question in QLQ PAN26. The sum score of all answers from all questions of QLQ PAN26 were correlated to the PACADI-score.

**Table 1:** Included questions from PACADI and QLQ PAN26

PACADI question	Relating questions from QLQ PAN26 version
Pain/discomfort	31: Have you had abdominal discomfort? 33: Have you had back pain? 34: Did you have pain during the night? 35: Did you find it uncomfortable in certain positions (e.g., lying down)?
Anxiety	41: Have you worried about your weight being too low? 51: Were you worried about your health in the future?
Loss of appetite	36: Were you restricted in the types of food you can eat as a result of your disease or treatment? 37: Were you restricted in the amounts of food you could eat as a result of your disease or treatment? 38: Did food and drink taste different from usual?
Itchiness	44: Have you had itching?
Fatigue	42: Did you feel weak in your arms and legs?
Dry mouth	43: Did you have a dry mouth?
Bowel and/or digestive problems	32: Did you have a bloated feeling in your abdomen? 39: Have you had indigestion? 40: Were you bothered by gas (flatulence)? 46: Did you have frequent bowel movements? 47: Did you feel the urge to move your bowels quickly?

#### Statistics and ethics

Correlation between PACADI and QLQ PAN26 was analyzed with Spearman’s correlation coefficient. Correlation was defined as: poor (*r* < 0.20), fair (*r* = 0.21–0.40), moderate (*r* = 0.41–0.60), good (*r* = 0.61–0.80) and very good (*r* = 0.81–1.00) [10].

The translation and validation were conducted in accordance with the ethical standards of the World Medical Association Declaration of Helsinki Ethical Principles for Medical Research Involving Human Subjects [11]. The regional Ethics Committee for the region of Västra Götaland, Sweden approved the study protocol. Patients received written information regarding the study also stating that consent was assumed on the return of a completed questionnaire.

## Results

In the translation process, three symptoms needed further discussions before a consensus could be made. The Norwegian word for anxiety can be translated to two different Swedish words. As anxiety can be both a symptom and a disease, we chose to include both words. The symptom “loss of appetite” in Swedish can mean both reduced and lost desire to eat. The former was chosen as it corresponds to clinical experience of patients' symptoms. A translation of the Norwegian word for fatigue into Swedish corresponds to “laxity” and was therefore not used. There is no direct translation into Swedish of the English word "fatigue". Two words were therefore decided to be able to cover all aspects of the symptom.

After the assessment of the preliminary version by health care personnel, suggestions were made to improve the questions. However, as the suggestions derived from the original Norwegian version no changes were made. Feed-back of the preliminary PACADI-version from patients did not reveal any suggestions for improvements in the instructions-, relevance-, descriptions- or response options of the items in PACADI. Reviewers all described PACADI as a good, relevant, and comprehensive instrument. However, reviewers did express concerns about the question on bowel and/or digestive problems in PACADI as it might be unclear and therefore confusing to the patients.

**Fig. 1:**
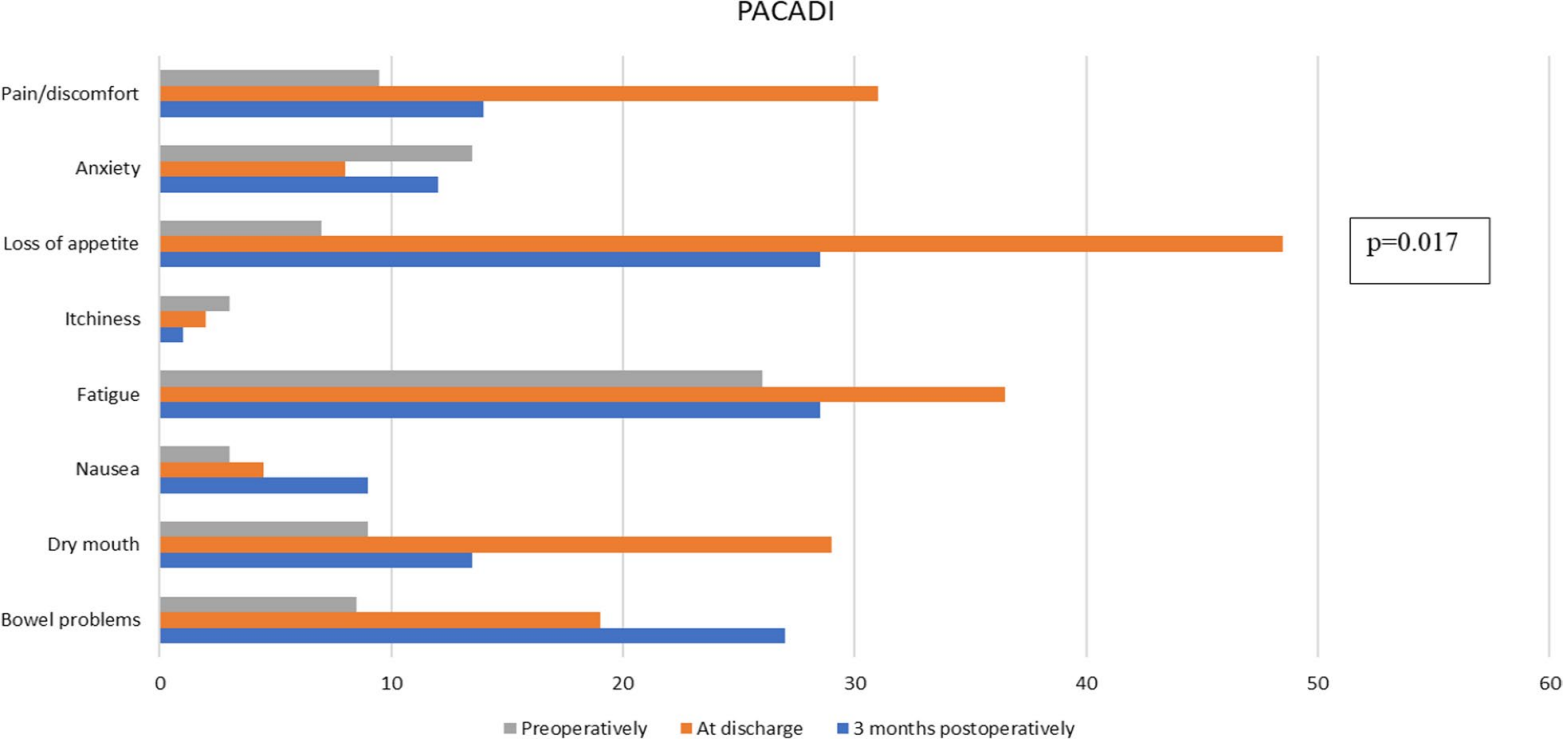
Results of the eight items in the questionnaire preoperatively, at discharge and three months postoperatively

The results of PACADI are given in Fig. 1. PACADI score was 0.166 preoperatively, 0.334 at discharge and 0.211 three months postoperatively (p = 0.71). Corresponding figures for PAN26 are 48.5; 59 and 50 (p = 0.229).

When exploring the correlation (Table 2) between PACADI and QLQ PAN26, there was a large diversity between the symptoms. One symptom (“fatigue”) correlated fairly (r_s_ 0.321) while four (“pain”, “anxiety”, “itchiness” and “bowel and/or digestive problems”) had moderate correlation (r_s_ 0.419–0.593). “Loss of appetite” and “dry mouth” had good correlation (r_s_ 0.682–0.717). The PACADI-score had a good correlation to the adjusted QLQ PAN26 sum score (r_s_ 0.709).

**Table 2:** Correlations between the PACADI and PAN-26

	Correlation coefficient
Pain/discomfort	0.572
Anxiety	0.419
Loss of appetite	0.682
Itchiness	0.593
Fatigue	0.321
Dry mouth	0.717
Bowel and/or digestive problems	0.579
Total scores	0.665
Adjusted total score PAN26 to PACADI	0.709

## Discussion

During the translation process three out of eight symptoms needed further discussions before consensus was reached. The English translation was taken into consideration during this process. In the decision of two of the elements the English version had an impact on the final wording. This has advantages as well as disadvantages. It is a risk that the translations built on each other rephrase the initial meaning, but in this case the English version would probably be the most used one and was therefore taken into consideration. In the guidelines used in the process [12] this handling is not included, but in future guidelines this may be involved as questionnaires are also developed in other countries with minority languages.

The patients were asked to assess the preliminary version of PACADI in writing. The patients did not suggest any improvements, nor did they point out any ambiguities, although the medical staff expressed some concerns regarding the question about "Bowel and/or digestive problems". However, this might reflect the comprehensive process during development of the questionnaire with involvement of both staff and patients. The group is large enough to evaluate the correlations between the questionnaires but has too few participants to analyze differences between the subgroups.

Both PACADI and QLQ PAN26 are questionnaires that measure symptoms and experiences over a period of one week. However, QLQ PAN26 is a multi-item scale measuring symptoms as a dimension in quality of life. In PACADI patients are plotting their symptoms on visual analogue scales from 0–10. The result is then weighted into a score for further analysis to capture the patients´ situation resulting in a PACADI score ranging from 0–100. QLQ PAN26 includes 26 questions which evaluate different symptoms and functions. The answers are given by using a four level Likert scale. Some symptoms are covered by one question, but some includes two or more questions to cover a broader perspective of the symptom. How comparable these questionnaires are, has not previously been evaluated.

The correlation between PACADI score and total score for PAN26 was good. However, there were variations in correlations of specific symptoms between PACADI and the related questions in PAN26. The differences may have several reasons, in PACADI the patients are asked for their experiences of a specific symptom in a single word. This opens up different ways of thinking about this word from a narrow to a broad spectrum. In the QLQ PAN26 questionnaire, three symptoms are covered by one question and the other ones by 2–5 different questions which may influence the results. The different correlation coefficient values can be explained by the lack of questions about the same symptom. For example, the symptom “Pain/discomfort” in PACADI is correlated with specific questions in QLQ PAN26 regarding pain in the back, pain at night, pain in specific positions or discomfort related to the taste of food. “Pain/discomfort” did correlate moderately between the two questionnaires. But pain can also be in other parts of the body, appear at other times than at night or even during other activities. This is the opposite in another symptom “Dry mouth”, where both questionnaires had similar questions and there was a “good” correlation between the two. One might speculate if a single question covering a symptom could be superior. However, “itchiness” includes only one question in QLQ PAN26, but it was only moderately correlated to the question in PACADI. Other factors such as differences in response alternatives (visual analogue scales vs. Likert scales) may have had an impact on the results. Another explanation could be that the questions compared are too dissimilar as “fatigue” and “weakness in legs and arms.

### Limitations

In the clinical part of this trial, the association between PACADI and QLQ PAN26 were evaluated in patients who were in different phases of their treatment. The patients were asked to complete the questionnaire at one of three different occasions, before surgery, at discharge or three months postoperatively. We have presented the results of PACADI for the whole group as well as subgroups depending on when the patients completed the questionnaire. Even though there are trends of differences in symptoms at different times they did not reach the level of significance except for “pain/discomfort” and “loss of appetite”. This is presumably a type two error and a limitation of the study. Another limitation is that not all patients did complete the questionnaire at every point in time. How different points in time affects the results has been evaluated in QLQ PAN26 [13]. The result indicated that the symptoms were worse post-operatively than at baseline, confirming the sensitivity of the QLQ PAN26 to detect clinically meaningful differences.

There is now a Swedish version of PACADI available to use in clinical practice. The initial validation showed a good correlation between PACADI score and adjusted total score PAN26, with good face validity. However, due to methodological limitations of this study there is a need for further studies of the of the psychometric properties in the Swedish version.

## Data Availability

Not applicable.

## Abbreviations

PACADI: Pancreatic cancer disease impact score
EORCT QLQ PAN 26: EORTC Quality of life Questionnaire—Pancreatic Cancer Module
PC: Pancreatic cancer
PROM: Patient reported outcomes
QoL: 0 Quality of life
EQ5-D: Euro QoL-5D
ESAS: Edmonton symptom assessment scale
